# Awareness of medical cannabis regulations among UK police officers – a cross-sectional study

**DOI:** 10.1177/00258172241237650

**Published:** 2024-05-17

**Authors:** Simon Erridge, Claire Wang, Lucy Troup, Mikael H Sodergren

**Affiliations:** 1 Medical Cannabis Research Group, Department of Surgery and Cancer, 4615 Imperial College London, UK; 2 Curaleaf Clinic, London, UK; 3 School of Education and Social Sciences, University of the West of Scotland, UK

**Keywords:** Cannabis, cannabidiol, tetrahydrocannabinol, police, law enforcement

## Abstract

Cannabis-based products for medicinal use were rescheduled in the UK in November 2018. The primary outcomes of this cross-sectional survey were to assess awareness of legislation governing these products among UK police officers and whether they had received appropriate training. 200 police officers completed the survey, and 57 (28.5%) respondents did not know these products were legal on prescription in the UK. 177 (88.5%) police officers believed they would benefit from more training on them and how to identify legal medical cannabis patients. Education on the legalities of cannabis-based products for medicinal use and why they are prescribed is necessary to improve knowledge among police officers.

## Introduction

In November 2018, the UK moved cannabis-based products for medicinal use in humans (CBPMs) to Schedule 2 under the Misuse of Drugs Regulations 2001,^1^ allowing unlicensed CBPMs to be prescribed. CBPMs may be prescribed only for individuals who have not sufficiently benefited or have been affected by intolerable side effects from licensed therapies for clinical conditions which may be amenable to treatment with CBPMs.^1^ These medications can only be initiated by a doctor listed on the General Medical Council’s Specialist Register with expertise in the condition for which CBPMs are being prescribed. The decision to prescribe must be confirmed by a multidisciplinary team containing doctors from other specialities.^1^

There were approximately 32,000 patients treated with CBPMs by the end of 2022 for conditions such as chronic pain, generalised anxiety disorder and post-traumatic stress disorder.^2^ Notwithstanding, it is estimated that 50% of the UK public are unaware that CBPMs can be prescribed legally.^3^ Consequently, whilst the number of patients prescribed CBPMs has continued to rise, these individuals continue to perceive themselves as being subject to stigma.^4^ In comparison to other groups, UK patients prescribed CBPMs are most concerned about what the police and criminal justice system may think about their medication.^4^ Stigma has been demonstrated to be both a cause and an exacerbating factor in driving health inequalities.^5^

Considering limited public awareness of legislation on CBPMs and the potential contribution of lack of police knowledge to perceived stigma among patients,^6^ this study’s primary aim was to assess the awareness of current legislation among UK police officers. Secondary aims included assessments of how much training was received on the topic and whether members of the police believed they had received sufficient training on CBPMs.

## Methods

A cross-sectional survey study was conducted between 24 October and 1 November 2022. The survey was designed by a multi-disciplinary team of academic clinicians and a cognitive neuroscientist with expertise in qualitative research.

Participants provided demographic information on sex, age and geographic location. The questionnaire contained the following questions:

1. Do you believe the following statement regarding cannabis is true or false? “Cannabis containing tetrahydrocannabinol (THC) can be fully legal when prescribed by an appropriate healthcare professional.”
2. Do you feel adequately educated on medical cannabis and the legalities around this prescription medicine?
3. Do you believe you and your colleagues would benefit from more training on medical cannabis and how to identify patients using legal prescription medical cannabis?
4. Have you ever encountered anyone, as part of your work, who claimed to be using cannabis containing THC on prescription?
5. (If yes to question 4) What did you do when encountering someone who claimed to be using cannabis containing THC on prescription?

The questionnaire was distributed to serving police officers in the UK by Opinium Research and weighted to derive a nationally representative sample. Data was analysed in Microsoft® Excel utilising descriptive statistics, except question five which was analysed utilising a thematic approach.

## Results

In total, 200 police officers (male n = 109; 54.5%) completed the survey. Ninety-six (48.0%) were between 18–34 years of age, another 96 (48.0%) between 35–54, and 8 (4.0%) were over 55.

Most respondents (n = 143; 71.5%) knew that cannabis is legal on prescription, whilst 42 (21.0%) and 15 (7.5%) participants thought that it was not legal or they were not sure.

**Figure 1.**
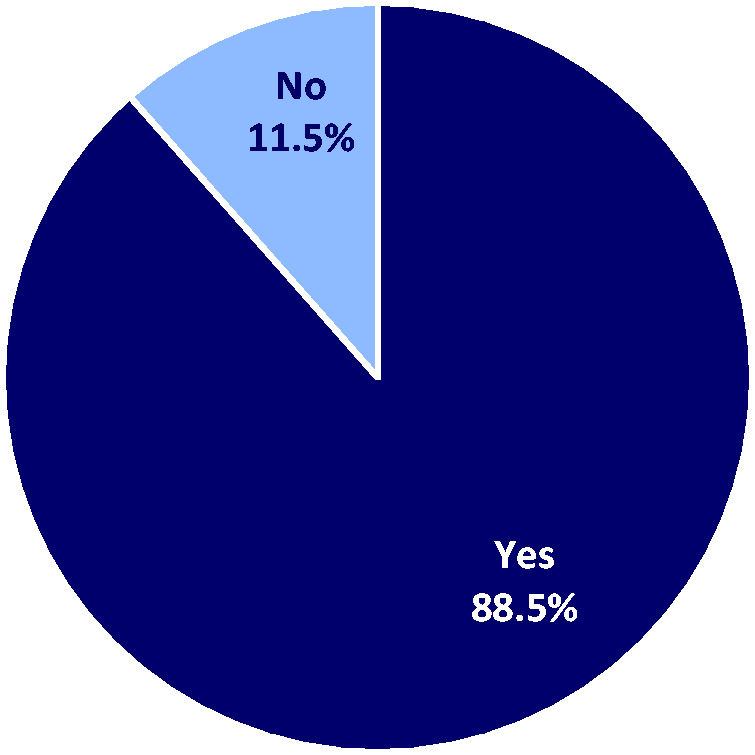
Responses to the question “Do you believe you and your colleagues would benefit from more training on medical cannabis and how to identify patients using legal prescription medical cannabis?” N = 200.

Forty-seven (23.5%) participants had never received formal training on this topic, whilst 85 (42.5%) had believed their received training was inadequate. Most police officers (n = 177; 88.5%) said they believed they would benefit from more training on CBPMs, including how to identify recipients of legally prescribed cannabis (Figure 1).

Eighty (40.0%) participants reported they had encountered someone during work who claimed to be using cannabis for medical reasons. The most common responses in thematic analysis of open answers detailing what action they subsequently took were asking for more evidence from the person themselves (n = 34; 42.5%), checking the legitimacy of their claim with a healthcare professional (n = 11; 13.8%), or asking for advice from a colleague (n = 8; 10.0%). Three (3.8%) respondents took no action at all. Two (2.5%) responses detailed the police officer giving advice to the individual in question. Six (7.5%) responses detailed the participant being detained or arrested, whilst two (2.5%) confiscated the individual’s cannabis. All other responses had independent themes and have been omitted to avoid invertedly de-anonymising respondents.

## Discussion

The results of this study show that a significant proportion (28.5%) of the police officers surveyed did not know the legal status of cannabis in the UK four years after the change in scheduling was implemented. Two-thirds of police officers had received either no or self-defined inadequate training on the legality of CBPMs. This gap in awareness can contribute to negative interactions between police officers and legitimate patients as evidenced by the thematic analysis of the respondents’ actions when they encountered a potential medical cannabis patient.

More than 1 in 5 police officers (21.0%) in this nationally representative survey believed that CBPMs were still illegal in the UK, even when prescribed by a medical doctor. A further 7.5% were not sure of their legal status. This is supported by responses suggesting that 66.0% of respondents had received inadequate training on CBPMs and the legalities of this medication class. There is limited comparable data from the UK or other jurisdictions; however, a 2022 paper from our group found that 84.4% of people in receipt of CBPM treatment feel stigmatised due to their medication.^4^ Studies from North America also highlight the reported stigma of medical cannabis patients.^6,7^ A common theme across these is how negative experiences with either the police or other aspects of the criminal justice system play a crucial role in this.

This is the first study which has aimed to assess police knowledge of CBPMs in the UK. A previous evaluation of the reclassification of illicitly sourced cannabis from a Class B to a Class C drug influenced the response of police officers towards individuals found in possession of cannabis.^8^ There is evidence of the reclassification of CBPMs having a similar effect, as the most common themes identified from the open responses to a question about how police officers had dealt with people who claimed to be using CBPMs were to appropriately seek more evidence from the person in question (42.5%) or a healthcare professional (13.8%).

Whilst this survey is derived from a nationally representative sample, there is no national police force in the UK. Instead, operational decisions regarding education and implementation of certain policies is the responsibility of the head of each police force area. Consequently, the sample size is insufficient to assess differences between police forces and identify the best performing regions.

This study ultimately highlights that there is insufficient knowledge on the legalities of CBPMs in the UK. Beyond this, even police officers who know CBPMs are legal when prescribed by a doctor have received insufficient education. As the number of patients treated with CBPMs continues to increase, it is imperative that police officers are provided with improved education at either a local or national level. This is likely to be best achieved by working in conjunction with regulated medical cannabis clinics and their specialist physicians.

## Data Availability

All data generated or analysed during this study are included in this article. Further enquiries can be directed to the corresponding author.

## References

[1] Case P. The NICE Guideline on medicinal cannabis: Keeping Pandora’s box shut tight? Medical Law Review 2020; 28(2): 401–411. 32016366 10.1093/medlaw/fwaa002

[2] Prohibition Partners. *The global cannabis report*, 3rd ed., https://prohibitionpartners.com/reports/the-global-cannabis-report-edition-3/ (2022, accessed 23 January 2024).

[3] Erridge S Coomber R Sodergren MH. Medical cannabis, CBD wellness products and public awareness of evolving regulations in the United Kingdom. Journal of Cannabis Research 2022; 4(1). DOI:10.1186/S42238-022-00165-6. 10.1186/s42238-022-00165-6 PMC9617440 36309761

[4] Troup LJ Erridge S Ciesluk B , et al. Perceived stigma of patients undergoing treatment with cannabis-based medicinal products. International Journal of Environmental Research and Public Health 2022; 19(12): 7499. 10.3390/ijerph19127499 PMC9223559 35742748

[5] Hatzenbuehler ML Phelan JC Link BG. Stigma as a fundamental cause of population health inequalities. American Journal of Public Health 2013; 103(5): 813. 23488505 10.2105/AJPH.2012.301069 PMC3682466

[6] Satterlund TD Lee JP Moore RS. Stigma among California’s medical marijuana patients. Journal of Psychoactive Drugs 2015; 47(1): 10. 25715067 10.1080/02791072.2014.991858 PMC4341951

[7] Bottorff JL Bissell LJL Balneaves LG , et al. Perceptions of cannabis as a stigmatized medicine: a qualitative descriptive study. Harm Reduction Journal 2013; 10(1): 2. 10.1186/1477-7517-10-2 PMC3584982 23414118

[8] Warburton H May T Hough M. Looking the other way: The impact of reclassifying cannabis on police warnings, arrests and informal action in England and Wales. British Journal of Criminology 2005; 45(2): 113–128.

